# Effect of Different Methods to Synthesize Polyol-Grafted-Cellulose Nanocrystals as Inter-Active Filler in Bio-Based Polyurethane Foams

**DOI:** 10.3390/polym15040923

**Published:** 2023-02-12

**Authors:** Dario Fontana, Federica Recupido, Giuseppe Cesare Lama, Jize Liu, Laura Boggioni, Selena Silvano, Marino Lavorgna, Letizia Verdolotti

**Affiliations:** 1 Chemistry Department, University of Pavia, Via Torquato Taramelli 12, 27100 Pavia, Italy; 2 Institute for Chemical Science and Technologies, CNR, Via Alfonso Corti 12, 20133 Milan, Italy; 3 Institute for Polymers, Composites and Biomaterials (IPCB-CNR), P.zzale Enrico Fermi 1, 80055 Portici, Italy; 4 State Key Laboratory of Polymer Materials Engineering, Polymer Research Institute of Sichuan University, Chengdu 610065, China

**Keywords:** CNCs, natural fillers, CNC dispersion functionalization, CNC solvent exchange-functionalization, bio-based polyurethane composite foams

## Abstract

Currently, the scientific community has spent a lot of effort in developing “green” and environmentally friendly processes and products, due the contemporary problems connected to pollution and climate change. Cellulose nanocrystals (CNCs) are at the forefront of current research due to their multifunctional characteristics of biocompatibility, high mechanical properties, specific surface area, tunable surface chemistry and renewability. However, despite these many advantages, their inherent hydrophilicity poses a substantial challenge for the application of CNCs as a reinforcing filler in polymers, as it complicates their dispersion in hydrophobic polymeric matrices, such as polyurethane foams, often resulting in aggregate structures that compromise their properties. The manipulation and fine-tuning of the interfacial properties of CNCs is a crucial step to exploit their full potential in the development of new materials. In this respect, starting from an aqueous dispersion of CNCs, two different strategies were used to properly functionalize fillers: (i) freeze drying, solubilization in DMA/LiCl media and subsequent grafting with bio-based polyols; (ii) solvent exchange and subsequent grafting with bio-based polyols. The influence of the two functionalization methods on the chemical and thermal properties of CNCs was examined. In both cases, the role of the two bio-based polyols on filler functionalization was elucidated. Afterwards, the functionalized CNCs were used at 5 wt% to produce bio-based composite polyurethane foams and their effect on the morphological, thermal and mechanical properties was examined. It was found that CNCs modified through freeze drying, solubilization and bio-polyols grafting exhibited remarkably higher thermal stability (i.e., degradation stages > 100 °C) with respect to the unmodified freeze dried-CNCs. In addition, the use of the two grafting bio-polyols influenced the functionalization process, corresponding to different amount of grafted-silane-polyol and leading to different chemico-physical characteristics of the obtained CNCs. This was translated to higher thermal stability as well as improved functional and mechanical performances of the produced bio-based composite PUR foams with respect of the unmodified CNCs-composite ones (the best case attained compressive strength values three times more). Solvent exchange route slightly improved the thermal stability of the obtained CNCs; however; the so-obtained CNCs could not be properly dispersed within the polyurethane matrix, due to filler aggregation.

## 1. Introduction

Currently, scientific research has focused on different processes as alternatives to the conventional treatments, i.e., synthesis and reprocessing of chemical substances, due to the concerns about environmental pollution, climate change and the shortage of resources. In this context, biomass wastes and by-products obtained from different industrial sectors have gained interest as sustainable source to produce chemicals that can be used (or re-used) in several industries, in spite of petroleum-derived chemicals [1,2].

Renewable resources provide the highest potential for solving the need for sustainability of the above-mentioned industries. Therefore, the development and commercialization of renewable raw materials have been intensified in the last decades. A variety of bio-based monomers, plastics, resins, fillers and additives are already commercially available. However, most of the currently available products still lack in mechanical or thermal performance due to a comparably high price and a limited life cycle [3].

One of the most petroleum-dependent sectors is plastic manufacturing, where one of the main products is polyurethane, representing about 7.8% of total traded plastics with the worldwide plastic production in 2020 of 367 million of tons [4].

In addition, polyurethane foams (PURs) are a significant subgroup of polyurethane materials that, due to their outstanding mechanical, chemical and physical properties, are used in a wide range of applications. Their applications include, but are not limited to, building and construction, thermal insulation, textiles, furniture, automotive, scaffolds for tissue engineering, refrigerators, wood substitutes and packaging [4,5,6]. Polyurethane foams are typically produced through polyaddition reaction between a polyol and a diisocyanate and a concomitant exothermal foaming reaction, where release of the expanding gasses occurs [5,6,7,8,9].

PUR foam precursors are traditionally derived from oil, but due to its status as a limited resource and environmental concern, biodegradable and renewable source-based raw materials are gaining remarkable attention. [9,10,11] In this regard, only a few research groups put their efforts to exploit renewable sources to synthesize sustainable isocyanates. [12,13] In contrast, several works have already been developing bio-sourced polyols [9,14,15,16] with the aim to replace those coming from petroleum-derived polyesters and polyethers [17]. As renewable sources, various types of vegetable oils are available, but the scarcity of hydroxyl groups (except for castor oil, which naturally contains an average of 2.7 hydroxyl groups per triglyceride, [18]) requires them to be modified.

The development of new bio-sourced fillers is desired not only for environmental issues but also because when used as reactive fillers in “partially sustainable” PU foams (made by conventional isocyanates and bio-sourced polyols), they impart comparable or higher thermal, mechanical and chemical–physical properties with respect to the oil-derived ones [19,20,21]. For instance, many are in studies in which PUR foam composites display good properties, such as flame retardancy [22,23], high flexibility, anti-aging [24,25], high impact strength and good thermal stability [26,27].

Among all possible fillers, cellulose is a natural carbohydrate-building block-material, obtained by several sustainable sources such as biomass [28], industrial paper and wastes [29]. In addition, cellulose is abundant, relatively low cost, non-toxic, biodegradable and a low-density material [30,31] whose dimensions range from the nano to micro scale.

In particular, cellulose nano crystals (CNCs) possess high mechanical strength, surface area, and aspect ratio [32]. Depending on its source, cellulose can be characterized by a different ratio between highly ordered-microfibrils, crystalline regions and comparatively less ordered-non-crystalline regions. Additionally, the extraction routes can influence the crystalline dimensions [32,33]. Several treatments, based on enzymatic, mechanical or chemical approaches, can isolate the crystalline cellulose microfibrils from the amorphous ones. Two are the most common chemical procedures: acid hydrolysis with sulphuric acid and oxidation with ammonium persulfate [34,35,36]. The CNCs obtained by these aforementioned approaches are used as filler in polymeric as well as in foam matrices to improve specific properties.

Nevertheless, cellulose is a hydrophilic filler; therefore, it is considerably difficult to mix such filler into the hydrophobic polyurethane matrix. Specifically, the filler dispersion in polymeric matrices is predominantly influenced by the interfacial and surface energy of the fillers. For this reason, the functionalization of CNC surface with specific functionalizers of different degrees of surface hydrophobicity broadens its field of application [37,38,39].

Various approaches are used to improve filler dispersion, e.g., interface modification, surface pre-treatment, use of coupling agents and incorporation of dispersants. By optimizing the performance of the interface, properties can be improved at both microscopic and macroscopic levels [40].

Therefore, designing and fabricating nanocomposites, where filler migration is discouraged (absence of agglomeration), is crucial. This can be accomplished by adding suitable functional groups, covalently bound on different polymer chains, enabling the reduction of the possibility of network rearrangement [41] or by opportunely functionalizing the filler surface [18,42].

In this latter case, several methods can be used for the filler surface treatment, based on bonding as well as on dispersing agents (namely compatibilizers) acting either through the reduction of the interfacial tension between fillers and polymer matrix or by reducing the filler-matrix interfacial energy, favoring filler dispersion [39,40,41,42,43]. Compared to other biopolymers used as fillers in composite polymer materials, i.e., lignin or starch, cellulose can be easily tailored to encourage its dispersion within polymeric matrices. On the contrary, lignin, which also possesses OH groups reacting with isocyanate components, displays some drawbacks such as poor solubility within organic solvents, high poly-dispersibility, poor reactivity due to the steric hindrance and brittleness and whose functionalization routes are still challenging and often require high energetic inputs [44].

On the other hand, inorganic fillers such as silica nanoparticles or clay-based bio fillers have been widely employed to produce composite PURs; however, without suitable surface functionalization, a phase separation (fillers/matrix) occurs inducing a worsening in final properties of the obtained foams [45].

Recently, both crystal and modified amorphous CNCs through ball-milling were employed within polyurethane foams to partially substitute OH groups of conventional polyols [46,47,48], producing a stiff polymer matrix, resulting in fine cell structure [46] and, in specific cases, improving electrical properties too [47]. Furthermore, CNCs were modified by means of organic solvents i.e., poly(ethylene glycol) [48], through silane chemistry. As a result, the obtained composite PURs showed a slight decrease in thermal conductivity and in compressive strength, due to the formation of partial open cells structure in the obtained foams.

This work is meant to be an extension of the work of Coccia et al. [1], where CNC_S_ were functionalized for the first time via suitable silanes-coupling agents to graft bio-derived polyol to be used as reactive fillers in flexible polyurethane foams tuning specific properties, such as thermal stability and compressive strength. This approach can be considered as a green approach as CNC modification occurs in an aqueous medium and under mild operative conditions [1]. Typically, CNCs possess three groups suitable for functionalization, although only one is actually available due to their steric hinderance. In this respect, four alkyl silanes were employed to create new reactive CNCs’ moieties available for polyol grafting, encouraging filler compatibility with PUR matrices. The best results in terms of polyol/silane grafting amount were attained by dispersing CNCs in dimethylformamide (DMF) and selecting trichloro(propyl)silane (TCPS) as a silane agent.

Improved thermal stability was attained by adding CNCs in polyurethane foams, while no important effect on PUR mechanical properties was recorded. To further improve the mechanical along with the thermal and functional properties of polyurethane foams, novel functionalization approaches were here investigated. More specifically, the CNC functionalization was carried out by grafting bio-based polyols with a silane-coupling agent, adopting different procedures than that reported in [1]. CNCs underwent two different initial treatments: (i) freeze-drying of the CNCs suspension (FD-CNCs) and (ii) solvent-exchange of the CNCs suspension (SE-CNCs). For both cases, solubilization of CNCs was conducted in DMA/LiCl system in place of the DMF reported in [1], due to better dispersion efficiency of the system as illustrated in [49,50] and subsequent polyol grafting using TCPS.

On the other hand, solvent exchange is also a valid green approach devoted to dispersing CNCs without altering their original crystallinity [51]. The effect of the two initial approaches on chemical and thermal degradation properties as well as on the functionalization degree of the fillers was evaluated. Additionally, at each given modification route, the effect of chemical nature of two commercial bio-based polyols on the modified CNCs characteristics was examined.

Subsequently, functionalized fillers were dispersed at 5 wt% in rigid bio-based polyurethane foams, and the effect on the thermal, mechanical and chemical properties of the produced bio-based composite foams were hence investigated for the first time. This is indeed worth mentioning that CNCs are usually employed in PUR matrices creating more open cell and softer structures [52]. The so-obtained filler functionalization led to good dispersion within the polyurethane matrix, preserving the closed cell-like morphology, typical of the rigid foams.

## 2. Materials and Methods

### 2.1. Materials

The bio-polyester polyols, obtained from renewable cashew nutshell liquid (CNSL), NX9201 (OH_n_ = 66 mg KOH/g, viscosity, η = 1.4 Pa∙s, average functionality = 2, bio-content of 87%) and NX9203 (OH_n_ = 94 mg KOH/g, η = 2.7 Pa∙s, average functionality = 2, bio-content of 69%) and used to graft CNCs powders, were purchased by Cardiolite Corporation (Bristol, PA, USA).

The bio-based branched polyether-polyester polyol BI6121(OH_n_ = 211 mg KOH/g, η = 2.5 Pa∙s, average functionality of 3.6, bio-content of 69%) was employed as a precursor during foaming. All the polyols were kindly provided by AEP Polymers (Basovizza, Trieste, Italy). Niax PM 40 and CH_3_COOK (Momentive S.r.l.,Termoli, Italy) were chosen as catalysts for the blowing and polymerization reactions, respectively.

The silicon surfactant L6900 (Momentive S.r.l.,Termoli, Italy), working as cell stabilizer, was purchased from Momentive S.r.l. Bi-distilled water (H_2_O) was used as blowing agent. Tris (2-chloroisopropyl)-phosphate (TCPP), designated as a flame retardant, was supplied by Evonik (Pandino, Italy).

MDI (Methylene diphenyl isocyanate) (SUPRASEC 2085), having 31.5% NCO-content, was purchased from Huntsman S.r.l. (Ternate, Italy).

The CNCs used in this work were supplied by MELODEA Ltd. (Rehovot, Israel) as a 3% aqueous suspension, consisting in a transparent gel. Particles exhibited size distribution in the range of 2–20 nm and length of 20–500 nm, respectively. Further details are already reported in [1].

N,N-dimethylacetamide (DMA) and Lithium Chloride (purchased by Sigma Aldrich, St. Louis, MO, USA) were used as the reaction solvent and catalyst to solubilize CNCs [48]. The silane n-butyl-trichlorosilane (TCPS, Sigma Aldrich) was selected as a silane agent.

Tetrahydrofurane (THF), acetone, methanol and ethanol (purchased by Sigma Aldrich, St. Louis, MO, USA) were used as solvents as received.

The TEA (triethylamine, purchased from Sigma-Aldrich, St. Louis, MO, USA), used as reaction solvent to dissolve CNCs, was distilled over calcium hydride before use hydride for at least 12 h prior to distillation under N_2_ flux (b.p. 88.8 °C/760 mmHg).

### 2.2. Preparation of Modified CNCs via Freeze-Drying and Solubilization in DMA/LiCl (FD-CNCs)

The aqueous CNCs suspension was lyophilized (LIO-5P lyophilizer, Trezzano Sul Naviglio, Italy) for 48 h at 0.205 mbar and −50 °C.

Subsequently, anhydrous DMA was added to the freeze-dried CNCs (FD-CNCs) until a concentration of 2.1 wt% of filler was obtained. The mixture was then heated at 160 °C for 1 h, then cooled down to 100 °C and then LiCl was added. The system was maintained at 100 °C, under vigorous stirring, until CNCs were completely solubilized and then added to the abovementioned solution, in order to attain the concentration of 7.6 wt%. The resulting solution was kept under agitation at the indicated temperature for 10 min as reported in [48].

Functionalization of FD-CNCs was conducted in two steps: first dissolution in TEA and then silanization. In details, 1.1 equivalent of TEA and n-butyl-trichlorosilane, respectively, were added and stirred for 2 h and 70 °C. Subsequently, 1 equivalent of bio-based polyol (properly solubilized in DMA under nitrogen flux) was added to the aforementioned colloidal solution for 3 h at 100 °C.

The reaction mixture was cooled to room temperature, and methanol was added to quench the non-reacted silane. All the modified CNCs were purified by unreacted polyols and/or silanes by using a Soxhlet apparatus with ethanol and then THF solvents. CNCs were then dried in an oven at 65 °C under vacuum.

**Scheme 1:**
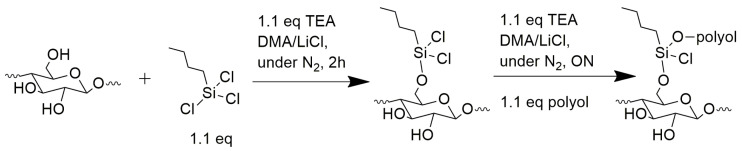
Mechanism of CNCs functionalization, with n-butyl-trichlorosilane in DMA/LiCl solvent reaction after freeze drying (FD_CNCs).

The general CNCs functionalization reaction mechanism in DMA/LiCl is reported in Scheme 1.

### 2.3. Preparation of Modified CNC via Solvent Exchange (SE_CNCs)

CNCs were functionalized according to the solvent exchange process. In details, acetone was added to the CNCs aqueous suspension in a ratio equal to 1:2 in volume and centrifuged for 5 min at 8000 rpm. Subsequently, the supernatant was removed, and the solid phase was rinsed out in acetone four times to assure that all water was removed. Then, DMA was added, and acetone was removed under vacuum conditions in a rotating evaporator at 50 °C and 40 mbar.

**Scheme 2:**
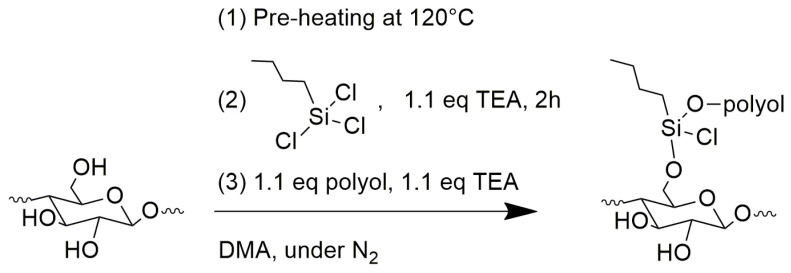
Mechanism of CNCs functionalization, SE-CNCs via solvent exchange procedure.

Afterwards, the CNCs were heated at 120 °C for 2 h. Silanization was, then, performed by adding to this suspension 1.1 equivalents of TEA and 1.1 of n-butyltrichlorosilane, respectively, and stirred for 2 h at 70 °C, according to the reaction reported in Scheme 2.

Next, 1.1 eq of TEA and 1.1 eq of polyol, properly solubilized in DMA under nitrogen flux, were added in the reaction batch. The reaction proceeded at 70 °C overnight.

The obtained functionalized CNCs are denoted as SE_CNCs.

For both the examined procedures, the bio-based polyols NX9203 and NX9201 were employed to graft CNCs, leading to four types of modified CNCs, reported as FD-CNC1, FD-CNC2, SE-CNC1 and SE-CNC2, respectively (Table 1). A comparison with unmodified freeze-dried CNCs and solvent exchange-CNCs, used as references, was conducted.

Therefore, the effect of different reaction media used to functionalize CNCs as well as of the grafting polyol chemical characteristics on chemical–physical and thermal properties of CNCs and of those of the obtained CNCs composite polyurethane foams were hence investigated.

In all cases, the functionalized fillers were dried at 80 °C under vacuum, before being used as fillers in PUR foams.

### 2.4. Preparation of Bio-Based PUR Foams

Bio-based PUR foams were prepared by using two steps-method. Initially, a solution was prepared (named as Component A) adding to polyol all the additives (e.g., catalysts, silicone surfactants, flame retardant and blowing agents, Table 2). Subsequently, a defined amount of CNCs was added to the mixture and vigorously mixed to well disperse the filler in Component A. Then, MDI (named as Component B) was added to Component A, by setting an NCO/OH ratio equal to 1.5. The obtained mixture was poured in a rectangular mold (20 × 10 × 4 cm^3^). The obtained composite PURs, corresponding to high density PU formulations according to [53], were cured for 3 h at 40 °C before performing any characterization.

In Table 2, the general composition of the obtained PUR foams for 100 g of total foam is reported, where, for all tested foams, 5 wt% of filler concentration was obtained. The selected formulation was considered according to the previous literature, where filler concentration was optimized [19]. Bio-based pristine polyurethane foams, i.e., foams without fillers, were considered as negative control. In addition, rigid PUR foam, reinforced with unmodified CNCs, was also obtained as proper comparison.

For the sake of brevity, CNCs-reinforced PURs are indicated as the following PUR pristine (no filler), PUR FD_0 (PUR reinforced with FD_CNC un-functionalized), PUR_SE_0 (PUR reinforced with SE_CNC un-functionalized), PUR_SE_1 (PUR reinforced with SE_CNC1), PUR_SE_2 (PUR reinforced with SE_CNC2), PUR_FD_1 (PUR reinforced with FD_CNC1) and PUR_FD_2 (PUR reinforced with FD_CNC2), respectively.

#### 2.4.1. CNCs and Polyols Characterizations

CNCs and grafting polyols were characterized by Fourier-Transform-Infra Red (FT-IR) Spectrometer Perkin Elmer (Waltham, MA, USA) in Attenuated Total Reflectance (ATR) mode from 650 to 4000 cm^−1^, 4 cm^−1^ resolutions and 64 scans.

Thermo-gravimetric (TGA, GA 7 Perkin Elmer (Waltham, MA, USA) analysis was carried out in a temperature range from 30 °C to 700 °C under nitrogen atmosphere to evaluate the amount of silane and polyols grafted on CNCs, respectively.

Low-magnification pictures of CNC particles were acquired with an optical microscope (model Z16 APO, Leica Microsystems GmbH, Wetzlar, Germany) and used to evaluate the mean particle size, by means of the tools provided by ImageJ software. (version 1.53 t, National Institute of Health, Bethesda, MD, USA).

#### 2.4.2. Bio-Based PURs Characterizations

FTIR analysis was conducted to verify the functional groups of the CNCs-composite PUR foams. FTIR spectra were recorded in the range of 650–4000 cm^−1^ wavenumber, using a resolution of 4 cm^−1^ and 32 scans.

Thermodegradative behavior of the selected bio-based PUR foams was assessed by means of thermogravimetric analysis (TGA, Q500 TA Instruments, New Castle, DE, USA) in the range of 30 °C to 600 °C under N_2_ flow (flow rate = 40 mL/min).

The thermal insulating property of bio-based produced PUR foams was assessed by measuring thermal conductivity at ambient conditions by using the modified transient plane source (MTPS) technique on a C-Therm TCi thermal conductivity analyzer (Fredericton, New Brunswick, Canada) [9,18]. The C-Therm analyzer was composed of a one-sided interfacial heat sensor (with a 17 mm of diameter) applying a momentary constant heat source to the sample with a measurement pulse between 1 and 3 s. Cylindrical foam samples (with diameter 6 cm and thickness equal to 5 cm) were obtained by coring from a larger panel. For each material, three specimens were selected and analyzed. These samples were carved out after one week from the foaming process, and the evaluation was carried out at room conditions (T = 25 ± 2 °C, RH = 40–50%).

Morphological analysis of composite PUR foams was carried out using SEM microscopy (SEM, FEI ESEM Quanta 200, operating at 30 kV using different magnifications). Foams were cut in the growth direction and coated with gold using a sputter coater (model SC500, emScope-now Quorum Technologies Ltd., Laughton, UK) before imaging. Cell size distribution and average cell size were evaluated using a commercial software for image analysis (Image J). For each tested foam, 50 entire cells were chosen to assure a statistically representative determination.

Compressive analysis of the composite foams was conducted by means of a universal testing machine (model 4304 from SANS, Shenzhen, China) with a calibrated 1 kN load cell. Cubic samples were cut from foamed slabs 55 mm × 55 mm × 30 mm in size according to the ASTM D1621 standard [54]. Compression tests were carried out at room temperature, and elastic modulus and compression strength were evaluated from compressive curves. For each sample, experiments were conducted at least in triplicate.

The obtained results were also studied by means of a modified Gibson–Ashby model. Starting from the well known equation [54] (Equation (1)), applied to closed-cells foams, when compression tests were performed and a linear elasticity behavior can be identified.

(1) $$\frac{E}{E_{S}}=∁\varphi^{2}{\left(\frac{\rho }{\rho_{S}}\right)}^{2}+C^{\prime }\left(1-\varphi \right)\left(\frac{\rho }{\rho_{S}}\right)$$

*E*/*E_S_* is known as relative modulus, given by the ratio between *E*, the foam modulus (expressed in MPa), and *E_S_*, the modulus of the cells material (set equal to 1600 MPa); *ρ*/*ρ_S_* is known as relative density, with *ρ* as the foam density (kg/m^3^) and *ρ_S_* as the density of the cells materials (set equal to 1200 kg/m^3^) [55]. The other symbols are *C* and *C*′, two proportionality constants and *φ* representing the solid volume fraction contained in cell edges (1 − *φ* is, then, the solid volume fraction contained in cell walls).

However, for *φ* = 1, so considering the foam made of open cells, where the all material is condensed on the edges, the Equation (1) becomes:

(2) $$\frac{E}{E_{S}}=∁{\left(\frac{\rho }{\rho_{S}}\right)}^{2}$$

From (2), *C* can be determined for each sample. Then, from this equation, a more general equation can be considered (3):

(3) $$\frac{E}{E_{S}}=∁{\left(\frac{\rho }{\rho_{S}}\right)}^{n}$$

already reported elsewhere [56], where the exponent *n* varies, depending on the analyzed foam, which can be determined when *C* = 1. [57]. From Equation (1), considering *φ* = 0, also *C*′ can be obtained for each foam.

In this regard, a more general equation can be written (4):

(4) $$\frac{E}{E_{S}}=∁\varphi^{2}{\left(\frac{\rho }{\rho_{S}}\right)}^{n}+C^{\prime }\left(1-\varphi \right)\left(\frac{\rho }{\rho_{S}}\right)$$

from the *φ* value of each foam was evaluated, providing a more complete description of the structure of the sample.

## 3. Results and Discussions

### 3.1. Filler Characterization

**Figure 1:**
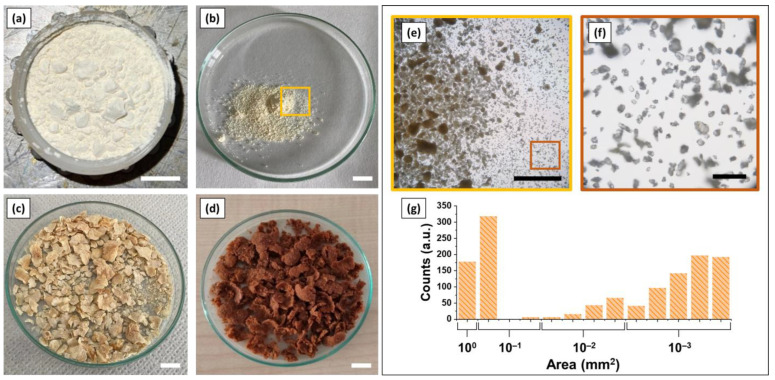
(**a**) FD_CNC1, (**b**) FD_CNC2 and (**c**) SE_CNC1 and (**d**) SE_CNC2. Scale bar is 1 cm for all pictures. (**e**) 2× and (**f**) 20× magnification of FD_CNC2, acquired with optical microscope. Scale bars are equal to 1 mm and 100 μm, respectively. (**g**) Average particle size distribution of modified CNCs.

Figure 1 displays the modified CNCs produced using freeze drying and via solubilization in DMA/LiCl, (FD_CNC1 (a) and FD_CNC2 (b), and those obtained through solvent exchange approach (SE_CNC1 (c) and SE_CNC2 (d)), respectively. NX9203 and NX9201 polyols were used to modify FD_CNC1, SE_CNC1 and FD_CNC2 and SE_CNC2, respectively.

In details, CNCs powders (FD_CNC1 and FD_CNC2, Figure 1a,b) were obtained by freeze drying and solubilization. On the contrary, the solvent exchange approach led to the flake-like formation, not easy to handle within the polyurethane matrix (Figure 1c,d). In this latter case, given its size and shape, a prior grinding was carried out to permit filler to be used in polyurethane formulations.

These results could be dictated by the different macromolecular structure of cellulose in the two different reactive media. In particular, for the FD_CNCs, as elsewhere analyzed [48], cellulose was totally destructured/dissoluted in DMA/LiCl cosolvent, for which the possible reaction mechanism can be here briefly described. The Cl^−^ anions form strong H-bonds with the CNCs hydroxyl protons. When cellulose is dissolved in DMA/LiCl, the Cl^−^ anions replace the OH···O hydrogen bonds between cellulose chains with the OH···Cl^−^ hydrogen bonds. As a consequence, the breakage of the cellulose intermolecular H-bonding as well as splitting of the Li^+^-Cl^−^ ion pairs occur [36]. Subsequently, the Li^+^ cations can be further solvated by free DMA molecules by enhancing the H-bonded Cl^−^ anions to reach electrical equilibrium [36]. In this way, cellulose chains can be easily dispersed at the molecular level in the solvent media leading to homogeneous solutions. Additionally, after silane functionalization and drying, in the obtained FD_CNCs powders, the singular cellulose molecules did not reassemble in aggregates (Figure 1a). On the other hand,the solvent exchange process seemed to induce aggregation of macromolecular CNC systems presenting a *flake-like* morphology.

Modified CNCs’ dimensions were evaluated by means of an optical microscope (Figure 1e,f), and image analysis procedures (Figure 1g). The powders particles presented variable sections, but mostly in two ranges, 0.5–1 mm^2^ and 1–10 μm^2^.

**Figure 2:**
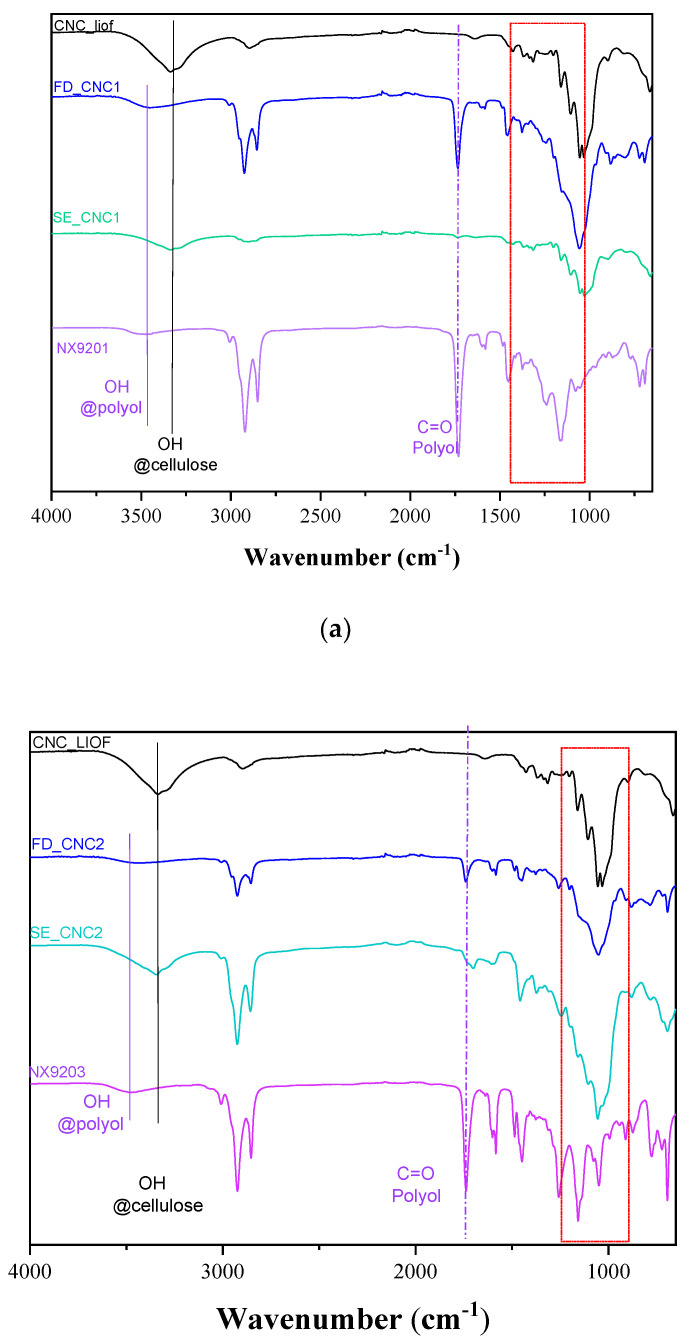
(**a**) FTIR spectra of CNCs modified with NX9203 polyol. (**b**) FTIR spectra of CNCs modified with NX9201 polyol. FTIR spectra in the range of 1500–1000 cm^−1^ are pointed out in the red box.

In Figure 2, ATR-FTIR spectra of the examined CNCs are reported, where differences in FTIR spectra of CNCs functionalized with NX9201 and NX9203 polyols are displayed in Figure 2a,b, respectively.

As observed, all the examined CNCs showed a broad band around 3200 cm^−1^ correlated to the OH-stretching (both inter- and intra-molecularhydrogen bonds in cellulose) and the characteristic stretching vibration of C-H around 2970–2900 cm^−1^. Furthermore, the wide bands in the range 1200–1000 cm^−1^ (depicted in the red box, Figure 2a,b), were correlated with the overlapping of several absortion vibrations, e.g., CH_2_ rocking, C-O-C and C=C stretching and the asymmetric in-plane ring stretching (~1053 cm^−1^), respectively [43,46,58]. Moreover, a decrease in OH peak intensity with respect to the freeze-dried CNCs (Figure 2a,b, black curve), as well as the appearing of the absorption peak of carbonyl C=O, correlated to the corresponding polyester polyol and could be observed for the case of modified freeze dried-CNCs (Figure 2a,b, blue curves).

Furthermore, the wide band centered around 1010–1180 cm^−1^ and shifted at higher wavenumbers, e.g., from 1022 cm^−1^ for pure freeze-dried CNCs to 1052 cm^−1^ for FD_CNC1 and FD_CNC2, respectively, Figure 2a,b due to the presence of polyol grafting to the CNCs. This outcome ensures that the functionalization occurred. Conversely, for the SE_CNC1 (Figure 2a, green curve), the absorption peak of carbonyl C=O, correlated to the polyester polyol did not appear, meaning that the functionalization did not take place. On the other hand, for the case of SE_CNC2 (Figure 2b, green curve), a slight peak around 1700 cm^−1^, associated with the C=O of polyol, could be observed, proving that a mild funzionalization with polyols occurred.

**Figure 3:**
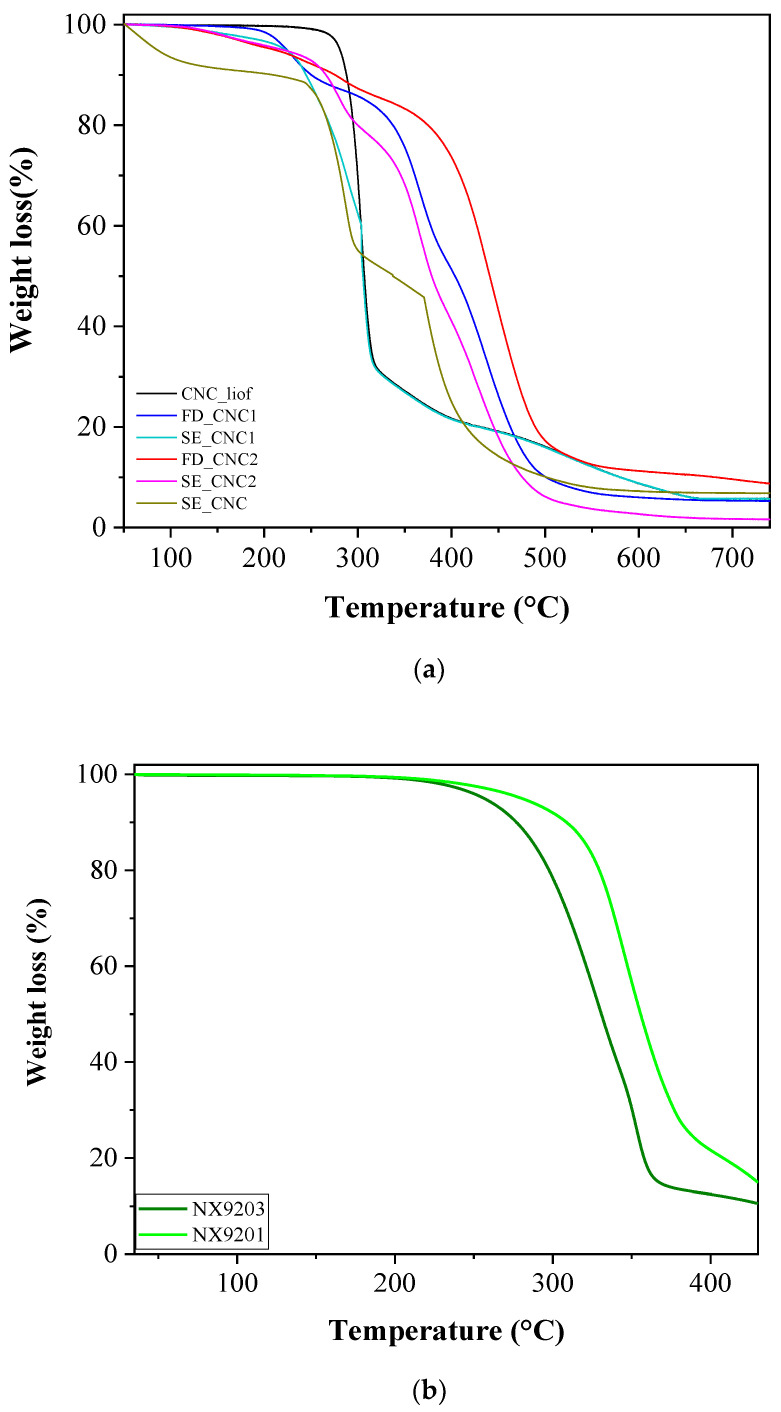
Thermogravimetric analysis (TGA) of the (**a**) CNCs fillers and (**b**) bio-polyols used for the functionalization.

TGA analysis was conducted to evaluate modified CNCs thermal stability by comparing with that of unfunctionalized CNCs. In Figure 3a, the thermograms of functionalized CNCs are reported. To appreciate the role of polyols in functionalizing CNCs, TGA measurements of the bio-based polyols are shown in Figure 3b.

For un-functionalized FD_CNCs, three degradation stages (Figure 3a, black curves) were recorded: one main degradation step occurring around 300 °C (with a weight loss of 70 wt%), due to the water evaporation and rearrangement of the CNCs, a second step (with a mass loss of around 10%) in the range 310–400 °C due to the degradation of CNCs linked to the sulphate groups (which were generated during the hydrolysis with sulphuric acid during the CNCs extraction process [58]) and a third peak of degradation due to the cross-bridging and aromatic cyclization of charred residue at 700 °C of around 8 wt%, respectively [58].

On the other hand, FD_CNC1 and FD_CNC2 (Figure 3a, blue and red curves) exibited a first step associated to the solvent and polyols degradation (~14 wt%) in the range 195–350 °C for the FD_CNC1 and 150–400 °C for the FD_CNC2, respectively, presenting similar thermodegradative behavior of NX9203 and NX9201 polyols, displayed in Figure 3b.

In addition, the degradation of the cellulosic products shifted to higher temperatures, i.e., 375 °C for FD_CNC1 and 460 °C for FD_CNC2 with respect to the unmodified FD_CNC (Figure 3a, black curve), which degraded at 300 °C, approximately. The difference in thermal stability of FD_CNC1 with respect to FD_CNC2 (around 100 °C) could be attributed to the higher thermal stability of NX9201 which affected positively the thermal stability of functionalized FD_CNC2. These outcomes can be compared with the recent literature where CNCs were modified through freeze drying, solubilization and silanization [1]. The selected functionalization route allowed the achievement of superior thermal stability behavior of the obtained CNCs with respect to the case of unmodified CNCs, while only the improvement of thermal stability at higher degradation temperature ranges could be identified by employing the functionalization route described in [1].

Instead, for the unfunctionalized SE_CNCs, the degradation steps occurred at lower temperature ranges compared with the un-functionalized FD CNCs as result of intercalation of high amount of solvent (acetone/water ~15 wt%) within the macromolecular structure. Conversely, the SE_CNC1 (Figure 3a, green curve), although obtained by solvent exchange route, showed a degradation behavior similar to the un-functionalized freeze-dried CNCs. More specifically, the first part of the degradation curve occurred at lower temperatures, as already explained, due to the evaporation of the solvents (i.e., water, acetone). The second part of degradation, associated with the CNC degradation, showed a similar trend to that of un-functionalized, freeze-dried CNCs.

Similar behavior was found for the case of SE_CNC2 (Figure 3a, pink curve). In details, a first degradation step (30–250 °C) due to the solvent evaporation (~6 wt%) as well as a second step degradation (250–310 °C) due to the NX9201 polyol decomposition (~11 wt%) (as also observed in Figure 3b which NX9201 polyol occured in the range 250–390 °C) was noticed. Finally, the third step was associated with the functionalized-cellulose decomposition (~77 wt%) that took place at higher temperatures (at about 60 °C more) with respect to both of the un-functionalized CNCs.

This could be perhaps dictated by crosslinking of CNCs with polyol. Finally, by analyzing the two procedures, it was possible to argue that the functionalization process using freeze drying and subsequent solubilization was more efficient, because both polyols were successfly grafted onto the CNCs surface, and the amount of grafted polyol was higher than that obtained using the solvent exchange approach.

In fact, on the basis of the equation reported in the work of Coccia et al. [1], in which briefly, from TGA analysis (achieved in N_2_), it was possible to determine the amount of grafted polyol on the CNCs by considering the final residue of the pristine and grafted CNCs after 400 °C (total degradation of cellulose). It was found that, the amount of sylane-polyols grafted to FD_CNCs was about double of those grafted on SE_CNCs (57 wt% vs. 25 wt%). Moreover, the amount of silane-polyol in FD_CNC1 was much higher than that of FD_CNC2 (57 wt% vs. 35 wt%).

### 3.2. Foam Characterization

Composite bio-based polyurethane foams were produced by adding 5 %wt of the examined CNCs within the polymeric matrix. The effect of the functionalization routes as well as of the chemical nature of the grafting polyols on foaming process as well as on the final characteristics of the obtained PUR foams was investigated.

**Figure 4:**
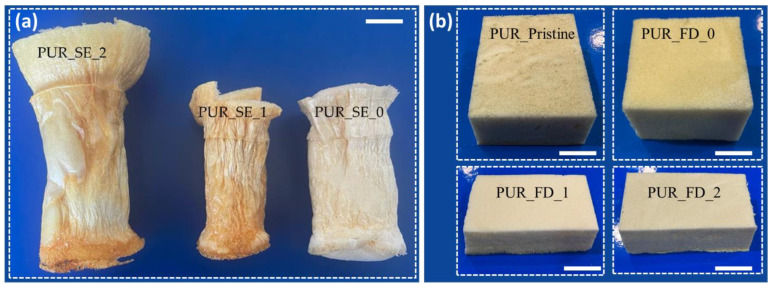
Digital reproductions of (**a**) PUR_SE_0, PUR_SE_1 and PUR_SE_2 foams, where CNCs were obtained through solvent-exchange procedure, and of (**b**) PUR_Pristine, and of PUR_FD_0, PUR_FD_1 and PUR_FD_2, for which CNCs were obtained through freeze-drying procedure. Scale bar = 1 cm.

PUR_SE_0, PUR_SE_1 and PUR_SE_2, produced through solvent exchange procedure are displayed in Figure 4. As observed, the addition of such fillers produced collapsed and shrinked foams (Figure 4a).

Such behavior might be due to a phase separation of the filler from the polymeric structure, that perhaps occured during the foam synthesis, which led to the cracking of a large part of the cells and subsequent collapse and shrinkage. In particular, it was noted that the addition of SE_CNCs fillers considerably accelerated the blowing reaction, achieving a remarkable temperature increase, not allowing both the polymerization and blowing to be easily controlled. This process strongly affected the distribution of the filler within the polyurethane matrix as well as its stability.

Conversely, no specific experimental difficulties were observed for the case of FD_CNCs, whose relative PUR foams are displayed in Figure 4b (lower panels) along with the pristine foams. In this respect, the effect of filler chemistry on PU foams characteristics were assessed. In details, PUR_FD_1 and PUR_FD_2 were obtained selecting two bio-based polyol-grafted fillers at the same composition (5 wt%). Therefore, the effect ot the two polyol chemistry on the dispersibility of the filler within polyurethane matrix and on final characteristics of the PUR foams was examined.

**Figure 5:**
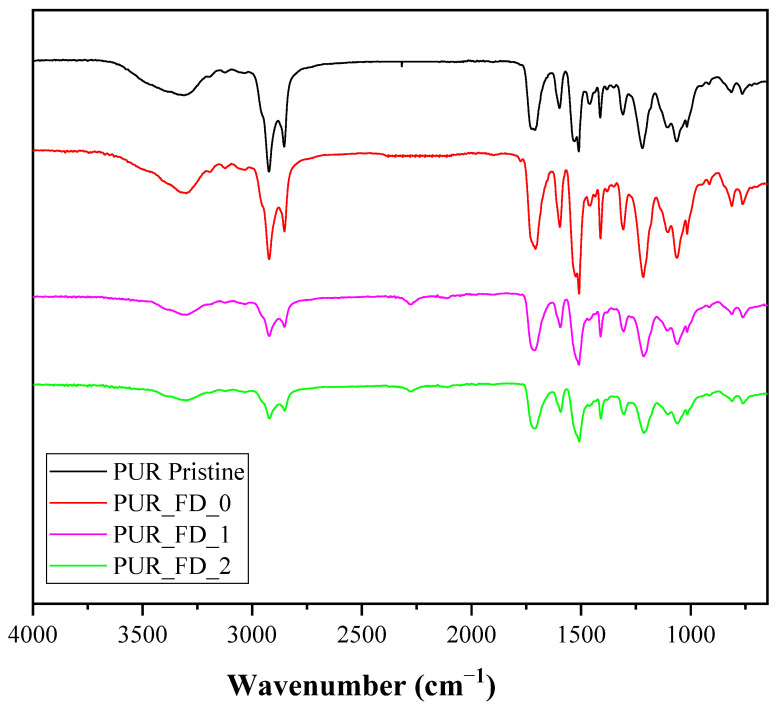
FTIR spectra of the composite PUR foams in the range of 3000–800 cm^−1^ wavenumber.

The occurring of the polymerization reaction was assessed by ATR-FTIR analysis (Figure 5). As observed, the absence of the NCO peak (at 2300 cm^−1^) associated with the occuring of polymerization reaction was detected along with the formation of urethane linkages. On the other hand, the vibration bands around 1700–1600 cm^−1^ (correlated to the carbonyl -C=O group), the carbamate species C-N at 1611 cm^−1^ and the ether –C-O-C of urethane groups around 100–1200 cm^−1^ confirmed the formation of urethane molecules. No significative differences were observed among the CNCs-composite PUR foams with respect to the pristine one.

**Figure 6:**
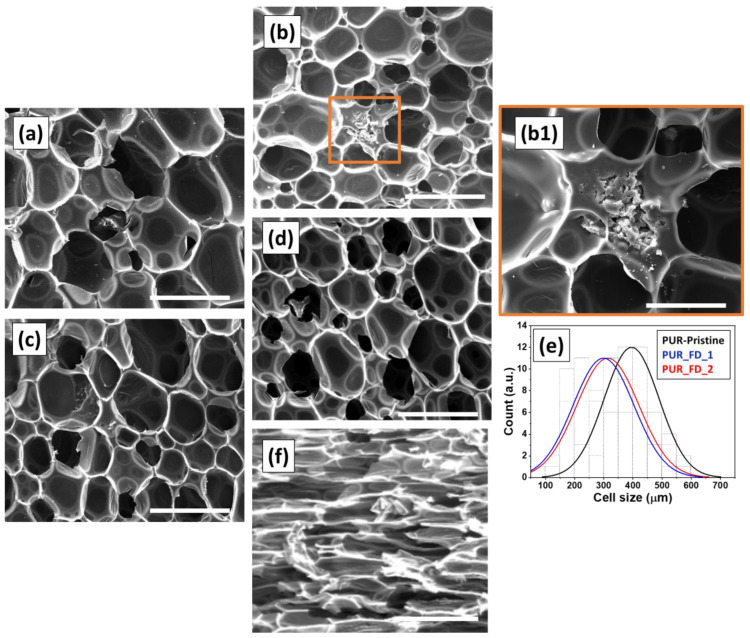
(**a**–**d**) SEM micrographs of PUR_Pristine, PUR_FD_0, PUR_FD_1, PUR_FD_2, respectively, at 200× magnification, scale bar = 500 μm; (**b1**) 500× Magnification of PUR_FD_0, scale bar 200 μm; (**e**) Cell size distribution of selected foams; (**f**) SEM micrographs of collapsed PUR_SE_1, 200× magnification, scale bar = 500 μm.

SEM micrographs of the foam cross sections are shown in Figure 6. For the case of solvent exchange route, it was decided to report PUR_SE_2 morphology to validate the occurring of shrinking and collapse of the produced foam. For the best examined cases (foams reinforced with FD_CNCs), cell size distribution was hence evaluated through image analysis and displayed in the lower part of Figure 6. This latter is a crucial factor dictating specific properties such as compressive strength or insulating capabilities of the obtained foams.

PUR pristine showed, as expected, a closed cell structure (typical of the rigid foams) [59] with a cell size ranging around 400 μm. For the cases of PUR_FD_0, PUR_FD_1 and PUR_FD_2, the addition of SF_CNCs induced a reduction of the average cell size, (around 100 μm for PUR_FD_1 and PUR_FD_2), with respect to the unfilled foam, where closed cell structure was preserved [6,9].

For the case of PUR_FD_0, it was possible to observe CNC aggregation (depicted in yellow circle in Figure 6), proving that CNCs were not appropriately dispersed with the polymer matrix. Similar results were observed also in the previous literature [17,60,61,62], where CNCs were selected as reactive fillers in rigid polyurethane foams leading to more closed cell structures with respect to the non-reinforced foam.

On the other hand, the effect of the filler functionalization seemed to be beneficial for CNC dispersion within the polyurethane matrix, as no cospicous agglomeration of CNCs was noticeable. In details, the intercalation of FD_CNC1 led to lower average cell size with respect to the case of PUR_FD_2, although showing similar size distribution. In this regard, it might be possible that the higher amount of the grafted bio-polyol along with greater OH number of the NX9203 polyol, used to graft FD_CNC1, played a key role in affecting filler dispersions and the final PUR foams characteristics.

These results were in agreement with the previous literature [1], where polyol-grafting CNCs were opportunely dispersed in a bio-based polyurethane matrix, leading to cell size reduction and improved interconnection within cellular structure, compared to the unfilled materials [1,53] due to the capability of CNCs to exfoliate and to react with isocyanate.

Finally, for the case of PUR_SE_2, shrinking along with a collapse of microstructure could be observed as not well filler dispersion within polyurethane matrix occurred and due to high reactivity of SE_CNCs, which might react extremely fast with isocyanate by forming urethane bonds around the filler, without expanding [63].

**Figure 7:**
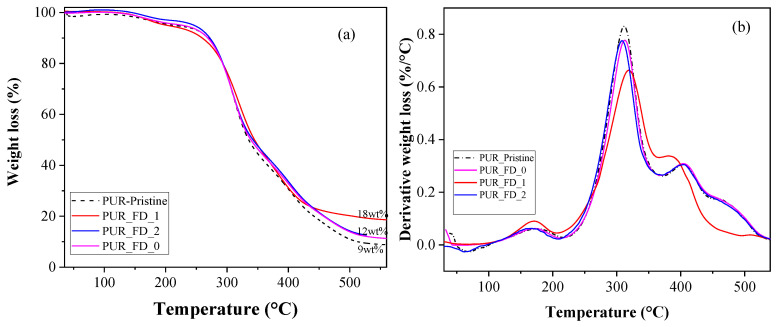
Thermogravimetric analysis (TGA) of the selected PUR foams. (**a**) TGA curves. (**b**) DTGA curves of the selected foams.

The thermodegradative behavior of the selected foams is shown in Figure 7. TGA and DTGA curves of the examined PUR foams are shown in Figure 7a,b.

Additionally, in this case, three main degradation stages with a corresponding weight loss percentages were noticed [1,6,9,22], e.g., first degradation stage, associated with the removal of low molecular weight-components, occurring in the range between 150 and 250 °C, then a second degradative stage associated mainly with the breakage of urethane linkages, followed by the formation of volatile molecules, in the range between 250 °C and 350 °C (maxima of degradation curves were observed at 300 °C, Figure 7b), and at higher temperatures, the degradation of components with more thermally stable bonds (i.e., aromatic rings present in the isocyanate and bio-based polyols). Furthermore, a residue of char was recorded at 600 °C in an inert atmosphere.

The examined composite bio-based PUR foams presented slightly higher degradation temperatures with respect to pristine PUR, with also a residual mass at 600 °C, higher than that of the unfilled system. This outcome might be associated with the particularly effective protection induced by the cellulosic filler, which translated into the ability to develop a protective, dense char during the thermal degradation process on the polyurethane matrix as already reported elsewhere [6,22]. However, it must be pointed out that the highest thermal stability was achieved for the case of PUR_FD_1, as it showed the highest mass residual at 600 °C. These findings are in agreement with [1].

Thermal conductivity of the examined PUR foams is reported in Table 3. Data are shown as average and standard deviation of three independent measurements, performed after 1 week from foam preparation.

The thermal conductivity of the selected foams was strongly affected by the cell size and distribution within the polyurethane matrix. In details, the reduction of cell dimension and high cell content associated with the nucleating effect of the modified CNCs induced reinforcement of cell walls of the polyurethane matrix, leading to better insulating performances [6]. The lowest thermal conductivity value was attained for the case of PUR_FD_1, as it exhibited the lowest cell size and distribution, which was found to be 30 % less than that of pristine and unmodified-CNCs reinforced PUR foams.

**Figure 8:**
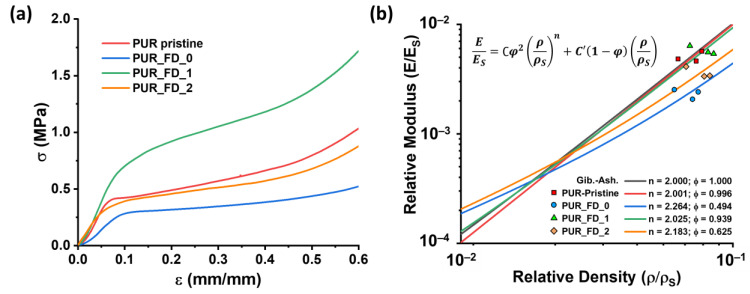
(**a**) Compressive stress vs. strain of the selected foams and (**b**) the resulting modified Gibson–Ashby model applied to the selected foams.

Mechanical properties were evaluated through compression test, and the resulting curves are reported in Figure 8.

Clearly, PUR_FD_1 showed higher mechanical properties with respect to the other foams, by presenting higher modulus and stress at 10% of deformation (Table 4), corresponding to the highest stress–strain curve among the present set of experiments. This sample had the same elastomeric behavior of PUR_FD_2, although this later revealed lower modulus and stress values. On the contrary, PUR-Pristine and PUR_FD_0, revealed behavior closer to an elastic-plastic one [64].

In general, the mechanical properties of foams, in terms of elastic modulus and stress values, were greatly influenced by cell morphology. Typically, these properties increased with decreasing cell size and increasing in closed pore content and vice versa, as widely studied by [65,66].

In the present case, these results could be easily attributed to the different interaction of each filler with the PUR materials, which produced an increase in average foam density with respect to the pristine and unmodified-CNCs reinforced PUR foams (Table 4). This aspect might be probably due to an increase in mixture viscosity due to functionalized filler intercalation [67]. In particular, when CNC_FD_0 was used, the material resultingly became weaker with respect to PUR-Pristine, from a mechanical point of view. This might be due to the fact that, when added to the mixture, its presence could reduce the good interaction between the reagents, and a phase separation (mostly due to the preferential interactions filler/filler) occurred, thus worsening the final polyurethane foam, since it created discontinuities in edges and walls of the cells of polyurethane. Then, when FD-CNCs were modified with NX9201, added to the Component A, further mixed with MDI and left to react, the mechanical properties of PUR-pristine seemed to be partially recovered, since the modified CNCs actively contributed to the load bearing. This contribution was fully exploited when CNCs were functionalized with NX9203, producing PUR_FD_1, for which the mechanical properties reached the highest value.

The improvement provided by PUR_FD_1 can be explained by the favorable interactions along with the filler-polyurethane interface. In this regard, polyol NX9203 provided a good reactivity to the fillers and an adequate interfacial interaction allowing a homogeneous dispersion in the matrix and an improvement in elastic modulus and in compression stress. Such mechanical behavior could be also explained by the reduction of the cell sizes, as observed from SEM images.

To better understand the mechanical behavior of the selected foams, a deeper analysis was necessary. Thanks to our modified Gibson–Ashby model, expressed with Eq. 4, it was possible to collect data on how much the rigidity was given by the edges or by the walls of the foam cells, thus giving a further insight of the interaction between matrix and the selected fillers.

To do so, Equation (4) becomes a second-degree equation, with φ as a variable. This Equation (5) was solved for each foam, by using E/E_S_, ρ/ρ_S_, n, C and C’ values (Table 5), obtained as described in Section 2.4.2, and final φ values were calculated.

(5) $$C{\left(\frac{\rho }{\rho_{S}}\right)}^{n}\varphi^{2}-C^{\prime }\left(\frac{\rho }{\rho_{S}}\right)\varphi +\left[\left(\frac{\rho }{\rho_{S}}\right)C^{\prime }-\frac{E}{E_{S}}\right]=0$$

The traditional Gibson–Ashby model, Equation (2), was usually considered to model the linear elasticity of open-cell foams, where all the solid material is condensed on the edges. Many polymer foams can be figuratively approximated [57] to this model, due to the fact that in most cases the cell walls resultingly are quite thin, presenting a weaker contribution to elasticity with respect to edges.

This last statement can be also conducted in the present case, where all samples exhibited thinner walls with respect to edges. In fact, even if SEM observation revealed a closed cell structure for all foams, the way the foams responded during a compression stress was more complex. Our model showed that the cell edges of each material contributed differently on the rigidity, depending on the chosen filler. In particular, as can be seen from Table 5, the volume fraction of PUR-pristine resulted quite close to 1, meaning that no discontinuities were present in the edges, which were practically bearing all the applied load and were negligible on the cell walls. Instead, for those samples with both unaltered and modified fillers, *φ* presented lower values. As can also be seen in Figure 8, PUR_FD_0 and PUR_FD_2 had the lowest volume fraction distributed on the edges, meaning that the contribution to the rigidity was extensively shared between edges and walls. Since the latter were quite thin, this determined a strong reduction in their mechanical properties. Moreover, PUR_FD_0 presented higher discontinuities on the edges volume, due to the low interaction with the filler. Instead, for PUR_FD_1, the higher mechanical properties were dictated by the strong solid material distributed along the cell edges, letting the cell walls not being involved in the foam rigidity, further proving the enhancement given by the presence of properly modified filler.

## 4. Conclusions

In this work, two different routes were selected to modify CNCs based on: (i) freeze drying followed by solubilization in DMA/LiCl systems (FD_CNCs) and (ii) solvent exchange (SE_CNCs), respectively. In all cases, silanization was conducted by grafting two different bio-based polyols. The effect of the modification procedures on the characteristics of CNCs was assessed.

It was found that CNCs obtained from freeze drying and solubilization presented remarkably higher thermal stability with respect to the un-functionalized, freeze drying CNCs (i.e., degradation temperature > 100 °C), whereas CNCs obtained from solvent exchange routes slightly improved their thermal stability with respect to the barely solvent-exchange fillers.

Functionalized CNCs were dispersed in bio-based polyurethane foams attaining different dispersion degrees of the filler according to the employed modification routes/grafting bio-based polyols. The best thermal characteristics were attained for the case of FD_CNC2. This might be ascribed to the larger amount of grafted polyol achieved upon silanization or due to the different functionalities.

However, it was not possible to disperse CNCs obtained using solvent exchange route (SE_CNC), within polyurethane foams, due to the occurring of phase separation within polymer structure, leading to collapse or shrinking of the obtained foams.

CNCs obtained via freeze drying/solubilization in DMA/LiCl systems could be effectively employed as reactive filler in polyurethane foams as they presented better dispersion within PUR foams with respect to the unfunctionalized CNCs, corresponding to more close cell-like structures and improved mechanical properties as well as higher thermal stability. The best mechanical performances were observed for the case of PUR_FD_1, where compressive strength is 50% and 60% more than those of pristine and unmodified CNC-reinforced materials, respectively. Additionally, in this case, the effect of the type of grafting polyols and their amount were crucial to achieving different CNC dispersion and therefore affecting the final characteristics of the composite PUR foams. Morphological quantitative parameters, estimated through the modified Gibson–Ashby model (i.e., n and φ) also supported this aspect, as PUR_FD_1 showed the most distributed material among cell edges which greatly allowed the bearing of the compressive load. As a future perspective of this work, further investigation of routes for CNC functionalization will be taken into consideration, with the aim to encourage CNC dispersion within the polyurethane matrix, including some upscaling possibilities. It might be crucial to investigate the effect of filler concentration on the composite PUR foam characteristics.

## Figures and Tables

**Table 1:** Description of modified CNCs.

Modified-CNCs	Polyol	Modification
FD_CNC1	NX9203	Freeze drying/Solubilization in DMA/LiCl/Sylanization
FD_CNC2	NX9201	Freeze drying/Solubilization in DMA/LiCl/Sylanization
SE_CNC1	NX9203	Solvent Exchange/Sylanization
SE_CNC2	NX9201	Solvent Exchange/Sylanization

**Table 2:** PURs formulations.

Components	PUR Pristine (%)	PUR_FD_0 (%)	PUR_SE_0 (%)	PUR_SE_1 (%)	PUR_SE_2 (%)	PUR_FD_1 (%)	PUR_FD_2 (%)
*Component A*							
BI6121	40.8	38.3	38.3	38.3	38.3	38.3	38.3
Niax PM 40	0.4	0.4	0.4	0.4	0.4	0.4	0.4
CH_3_COOK	0.4	0.4	0.4	0.4	0.4	0.4	0.4
Niax L6900	0.4	0.4	0.4	0.4	0.4	0.4	0.4
TCPP	4	4	4	4	4	4	4
H_2_O	0.4	0.4	0.4	0.4	0.4	0.4	0.4
Filler *	-	5.1	5.1	5.1	5.1	5.1	5.1
*Component B*							
MDI	53.5	51.0	51.0	51.0	51.0	51.0	51.0

**Table 3:** Thermal conductivity of the selected foams.

Samples	λ (W/mK)
PUR pristine	0.039 ± 0.002
PUR_FD_0	0.041 ± 0.005
PUR_FD_1	0.027 ± 0.007
PUR_FD_2	0.034 ± 0.009

**Table 4:** Mechanical properties upon compression tests.

	σ (10%) (MPa)	E (MPa)	ρ (kg m^−3^)	σ (10%)/ρ (MPa/kg m^−3^)	E/ρ (MPa/kg m^−3^)
PUR pristine	0.42 ± 0.03	8.03 ± 0.53	85.19 ± 3.02	4.93	94.27
PUR_FD_0	0.28 ± 0.02	3.74 ± 0.16	82.57 ± 1.11	3.39	45.29
PUR_FD_1	0.70 ± 0.05	9.26 ± 0.41	94.19 ± 3.97	7.43	98.31
PUR_FD_2	0.39 ± 0.04	5.77 ± 0.39	91.18 ± 4.13	4.28	63.28

**Table 5:** Outcomes from Gibson–Ashby model of studied foams.

Material	E/E_S_	ρ/ρ_S_	n	C	C’	φ
PUR_Pristine	0.0050	0.0710	2.0015	1.0041	0.0707	0.9959
PUR_FD_0	0.0023	0.0688	2.2637	2.0255	0.0340	0.4937
PUR_FD_1	0.0058	0.0785	2.0246	1.0646	0.0737	0.9393
PUR_FD_2	0.0036	0.0760	2.1826	1.6009	0.0475	0.6247

## Data Availability

Data sharing is not applicable for this article.
